# Quantitative assessment of brown adipose tissue metabolic activity and volume using ^18^F-FDG PET/CT and β3-adrenergic receptor activation

**DOI:** 10.1186/2191-219X-1-30

**Published:** 2011-12-01

**Authors:** M Reza Mirbolooki, Cristian C Constantinescu, Min-Liang Pan, Jogeshwar Mukherjee

**Affiliations:** 1 Preclinical Imaging Center, Department of Psychiatry and Human Behavior, University of California-Irvine, Irvine, CA, 92697, USA

**Keywords:** BAT, CL316,243, β3-adrenoceptor, ^18^F-FDG, obesity

## Abstract

**Background:**

Brown adipose tissue [BAT] metabolism *in vivo *is vital for the development of novel strategies in combating obesity and diabetes. Currently, BAT is activated at low temperatures and measured using 2-deoxy-2-^18^F-fluoro-D-glucose [^18^F-FDG] positron-emission tomography [PET]. We report the use of β3-adrenergic receptor-mediated activation of BAT at ambient temperatures using (*R, R*)-5-[2-[2,3-(3-chlorphenyl)-2-hydroxyethyl-amino]propyl]-1,3-benzodioxole-2,2-dicarboxylate, disodium salt [CL316,243] (a selective β3-adrenoceptor agonist) and measured by ^18^F-FDG PET/computed tomography [CT].

**Methods:**

Control and CL316,243-treated (2 mg/kg) male Sprague-Dawley rats were administered with ^18^F-FDG for PET/CT studies and were compared to animals at cold temperatures. Receptor-blocking experiments were carried out using propranolol (5 mg/kg). Dose effects of CL316,243 were studied by injecting 0.1 to 1 mg/kg 30 min prior to ^18^F-FDG administration. Imaging results were confirmed by autoradiography, and histology was done to confirm BAT activation.

**Results:**

CL316,243-activated interscapular BAT [IBAT], cervical, periaortic, and intercostal BATs were clearly visualized by PET. ^18^F-FDG uptake of IBAT was increased 12-fold by CL316,243 vs. 1.1-fold by cold exposure when compared to controls. ^18^F-FDG uptake of the CL-activated IBAT was reduced by 96.0% using intraperitoneal administration of propranolol. Average ^18^F-FDG uptake of IBAT increased 3.6-, 3.5-, and 7.6-fold by doses of 0.1, 0.5, and 1 mg/kg CL, respectively. *Ex vivo *^18^F-FDG autoradiography and histology of transverse sections of IBAT confirmed intense uptake in the CL-activated group and activated IBAT visualized by PET.

**Conclusion:**

Our study indicated that BAT metabolic activity could be evaluated by ^18^F-FDG PET using CL316,243 at ambient temperature in the rodent model. This provides a feasible and reliable method to study BAT metabolism.

## Introduction

The brown adipose tissue [BAT] plays a critical role in regulating body fat stores and may hold promise in combating obesity and diabetes given its extraordinary metabolic capacity [1,2]. Brown adipocytes are rich in mitochondria with densely packed cristae expressing uncoupling protein-1 [UCP-1]. They use lipids and carbohydrates to generate heat by uncoupling electron transport from oxidative phosphorylation [3]. The physiologic consequence of this function is an unrestrained oxidation by drawing lipids and carbohydrates from outside the cell [4]. BAT has also been found to be important in understanding the mechanism of insulin resistance [5], reducing adiposity, and improving type 2 diabetes [6] which make it a valuable target to study the pathogenicity of obesity and diabetes.

**Figure 1:**
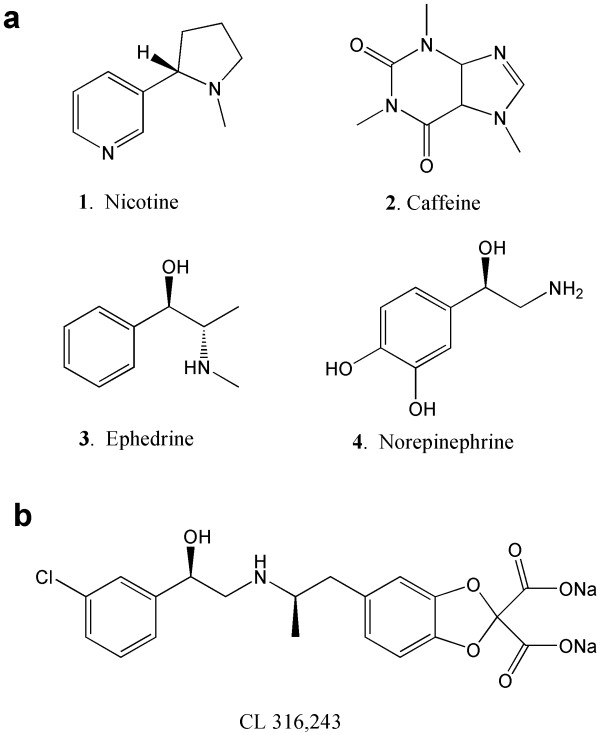
**Chemical structures of compounds used to activate BAT and measured by ^18^F-FDG**. Nicotine (1), caffeine (2), ephedrine (3), and norepinephrine (4) (**a**) and CL 316,243 (**b**).

Measuring the metabolic activity of BAT and assessing the factors that influence BAT activity are important for the development of novel strategies in the regulation of body weight. BAT is active when its thermogenic function is stimulated [7], and accumulation of 2-deoxy-2-^18^F-fluoro-D-glucose [^18^F-FDG] is a direct consequence of BAT activity [8]. Metabolic imaging of BAT using ^18^F-FDG positron-emission tomography [PET] was first reported in a cold-activated BAT in rodents [9]. Consequently, several ^18^F-FDG PET studies have been performed in the cold in both humans (approximately 16°C) [10] and rodents (approximately 4°C) [11] with some degree of success [12]. In order to activate BAT at ambient temperatures, several pharmacological challenges have been reported [13,14]. These include nicotine, caffeine, ephedrine, and norepinephrine, all of which directly or indirectly stimulate the noradrenergic receptors (Figure 1a).

**Figure 2:**
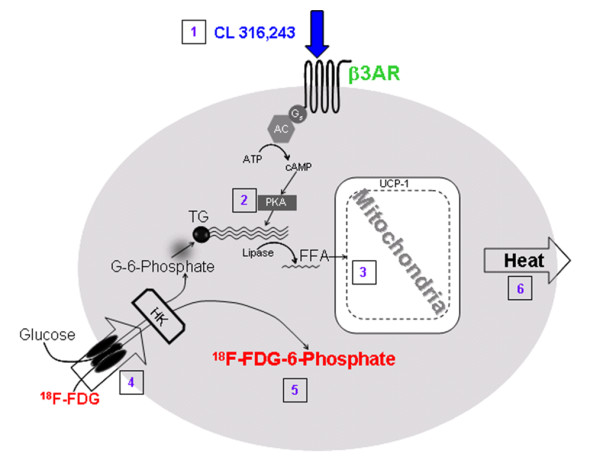
**A schematic showing the activation of a brown adipose cell**. (1) Agonists (norepinephrine and CL316,243) activate the β3-AR. (2) Production of cyclic adenosine monophosphate (cAMP) and protein kinase A (PKA) triggers lipase and hydrolysis of triglycerides. (3) The UCP-1 in the mitochondria increases the breakdown of fatty acids in the cell. (4) Increased metabolic activity in the mitochondria increases glucose and ^18^F-FDG uptake. (5) ^18^F-FDG-6-phosphate uptake in the cell increases and is measured by PET. (6) Outcome of this cascade of events is the formation of heat.

β3-Adrenergic receptors [β3-AR] are found predominantly on brown adipocytes [15]. BAT is innervated by the sympathetic nerves containing norepinephrine which activates β3-AR (Figure 2). Exposure to cold temperatures may promote metabolism indirectly through the activation of β3-AR [16]. Efforts have been made to develop β3-AR-selective agonists as possible therapeutic agents for the treatment of obesity. (*R, R*)-5-[2-[2,3-(3-chlorphenyl)-2-hydroxyethyl-amino]propyl]-1,3-benzodioxole-2,2-dicarboxylate, disodium salt [CL316,243] (Figure 1b) is a β3-AR-selective agonist. Binding affinity of CL316,243 is low for the β1- and β2-ARs, thus exhibiting a > 10,000-fold selectivity (binding affinities of CL316,243: Ki: β1 ≥ 10^-4 ^M; β2 = 3 × 10^-5 ^M; β3 = 3 × 10^-9 ^M) [17,18].

Chronic CL316,243 administration has been shown to have an antiobesity effect in mice and rats [19,20]. Direct evidence on the ability of acute β3-AR stimulation by CL316,243 to increase BAT metabolism *in vivo *using PET has not been reported. Our goal in this study was to quantitatively analyze ^18^F-FDG uptake in rats treated with CL316,243 using PET and computed tomography [CT]. The studies performed included the (1) measurement of acute effects of CL316,243 to activate and quantitate different BAT regions in the normal rat at ambient temperature using ^18^F-FDG uptake; (2) evaluation of dose effects of CL316,243 in BAT activation; (3) comparison of ^18^F-FDG uptake in cold temperature and propranolol (a β-AR antagonist) in CL316,243-treated rats; and (4) evaluation of BAT histology of the control and CL316,243-treated rats.

## Materials and methods

### General methods

Radioactivity was counted using a CRC-R dose calibrator (Capintec, Inc., Ramsey, NJ, USA), while low-level counting (< 60 kcps) was done using a Caprac-R well counter (Capintec, Inc.). An Inveon dedicated PET scanner (Siemens Medical Solutions, Inc., Malvern, PA, USA), which has a resolution of 1.46 mm in the center of the field of view [21], was used for the PET studies. An Inveon Multimodality [MM] CT scanner (Siemens Medical Solutions, Inc.) was used for CT acquisitions in combined PET/CT experiments. All *in vivo *and *ex vivo *images were analyzed using ASIPro VM (Siemens Medical Solutions, Inc.), PMOD Software (PMOD Technologies Ltd., Zurich, Switzerland), and Inveon Research Workplace [IRW] software (Siemens Medical Solutions, Inc.). Slices of BAT were prepared using the CM1850 cryotome (Leica Microsystems Inc., Buffalo Grove, IL, USA). *Ex vivo *^18^F-FDG-labeled sections were exposed to phosphor films and read using the Cyclone Phosphor Imaging System (Packard Instruments, Meriden, CT, USA) and were analyzed using the OptiQuant software (Packard Instruments). All animal studies were approved by the Institutional Animal Health Care and Use Committee of the University of California-Irvine.

### Animals

Male Sprague-Dawley rats, aged 11 to 12 weeks, weighing 343 ± 9 g at the beginning of the experiment, were used in this study. The rats were purchased from Harlan Laboratories, Inc. (Placentia, CA, USA) and housed under controlled temperatures of 22°C ± 1°C in a 12-h light-dark cycle, on at 6 a.m., with water and food chow *ad libitum*.

### Experimental protocol

#### General procedures

All rats were fasted for 24 h before ^18^F-FDG administration. The control, cold-exposed, and CL316,243- (Tocris Bioscience, Ellisville, MO, USA) and propranolol-pretreated (Sigma-Aldrich Corporation, St. Louis, MO, USA) rats were administered intravenously [i.v.] with 14 ± 1.5 MBq ^18^F-FDG under 2% isoflurane anesthesia. Following the injections, the rats were awake for 60 min and subsequently placed in the supine position in a rat holder and anesthetized with 2% isoflurane for upper-body PET imaging. The rat holder was placed on the PET/CT bed, and all animals had a CT scan after the PET scan for attenuation correction and anatomical delineation of PET images.

#### Drug effects

The control and CL316,243-pretreated rats (CL316,243, 2 mg/kg i.v., 30 min before ^18^F-FDG administration) were anesthetized for upper-body PET imaging (*n *= 3, each group). The same method of injection of ^18^F-FDG was used for all rats, as was the recovery period and re-anesthetization for PET. Dose effects of CL316,243 were investigated by injecting 0.1, 0.5, and 1 mg/kg of CL316,243 30 min prior to ^18^F-FDG administration. To evaluate whether enhanced ^18^F-FDG uptake in activated BAT could be reduced by pharmacologic interventions, 5 mg/kg propranolol was given intraperitoneally in the anesthetized rats 30 min prior to CL316,243 administration. Immediately after PET imaging, the rats were sacrificed; BAT and white adipose tissue [WAT] were harvested for autoradiography and counted in the well counter.

#### Temperature effects

Cold-exposed rats (*n *= 3, each group; cold treatment was at 8°C for 120 min prior to PET imaging) were administered i.v. with ^18^F-FDG under 2% isoflurane anesthesia. During cold exposure, the rats were caged alone without any sand bedding. Following the injections, the rats were awake for 60 min at ambient temperature (for control) and 8°C (for cold treatment) and subsequently anesthetized with 2% isoflurane for PET imaging.

### ^18^F-FDG PET/CT imaging

The Inveon PET and MM CT scanners were placed in the 'docked mode' for combined PET/CT experiments. After 60 min of ^18^F-FDG uptake, the rats were anesthetized and placed in supine position with their chest, neck, and head within the field of view of the PET scanner. PET data were acquired for 30 min, followed by a CT scan (large area detector, 10 cm × 10 cm field of view) for attenuation correction and anatomical delineation of PET images. The CT projections were acquired with the detector-source assembly rotating over 360° and 720 rotation steps. A projection bin factor of 4 was used in order to increase the signal-to-noise ratio in the images. The CT images were reconstructed using cone-beam reconstruction with a Shepp filter with a cutoff at the Nyquist frequency and a binning factor of 2, resulting in an image matrix of 480 × 480 × 632 and a voxel size of 0.206 mm. The PET images were spatially transformed to match the reconstructed CT images. PET images were corrected for randoms, attenuation, and scatter and were reconstructed as 128 × 128 × 159 matrices with a transaxial pixel of 0.776 mm and a slice thickness of 0.796 mm using a fast maximum *a posteriori *[fastMAP] algorithm in conjunction with three-dimensional ordered subset expectation maximization [OSEM3D] (2 OSEM3D iterations, MAP 18 iterations, 0.1 smoothing factor). All images were calibrated in units of becquerels per cubic centimeter by scanning a ^68^Ge cylinder (diameter 6 cm) with a known activity and reconstructing the acquired image with parameters identical to those of ^18^F-FDG images.

### Image analysis

#### ^18^F-FDG quantitation

The magnitude of BAT ^18^F-FDG activation was expressed as the standard uptake value [SUV] which was defined as the average ^18^F-FDG activity in each volume of interest [VOI] (in kilobecquerels per cubic centimeter) divided by the injected dose (in megabecquerels) times the body weight of each animal (in kilograms). The SUVs were thus expressed in units of [kilobecquerels per cubic centimeter per (megabecquerel per kilogram)].

#### Volume measurement of BAT

**Figure 3:**
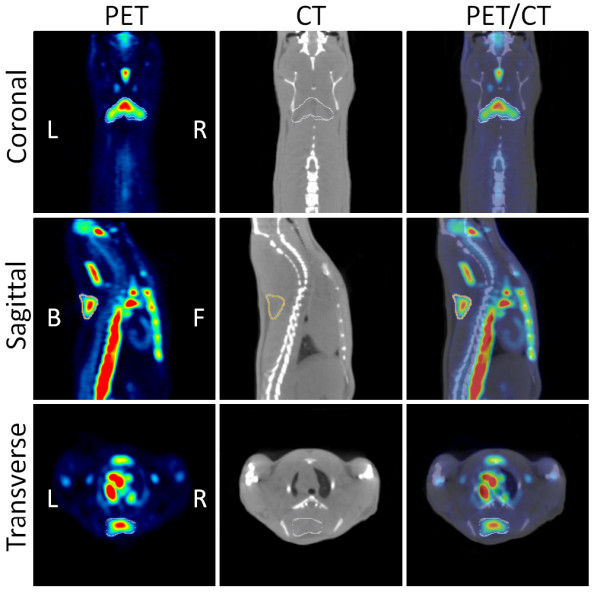
**Coronal, sagittal, and transverse views of PET/CT images**. The images show how automatic contour VOIs were drawn on the CL316,243-activated BATs: PET (left), CT (middle), and fused PET/CT (right).

The interscapular, cervical, periaortic, and intercostal BAT were visualized on 3-D CT images with the 3-D Visualization toolbox of the IRW software. For quantitative analysis, VOIs were drawn on PET images using PMOD. Similar to as described previously [22], the VOIs were first delineated visually by contouring the ^18^F-FDG activity that was clearly above the normal background activity (Figure 3).

In order to measure the volume of activated BAT, a new VOI contour was delineated based on a threshold equal to the mean ^18^F-FDG activity minus one standard deviation of all voxels within the primary visually defined VOI. The volume of the newly delineated VOI was used to report the activated BAT volume.

### Autoradiography

Transverse sections obtained from the soft tissue of the interscapular BAT [IBAT] region at the level of T5 were frozen on dry ice to provide 60-μm-thick slides for autoradiography. The slides were exposed to phosphor screens for 1 h and scanned using the Cyclone Plus storage phosphor system. The acquired image was then analyzed with the OptiQuant™ software where data in each ROI were quantified in digital light units per millimeter squared [DLU/mm^2^].

To confirm IBAT activation, biopsies were taken for histological evaluation (hematoxylin and eosin [H&E] staining) using 20-μm-thick frozen sections. Samples were viewed by light microscopy to determine the presence of multilocular droplets. Images were saved as TIFF files (16 bits) and were quantified using the ImageJ software for light intensity (range 0 to 256).

### Statistical analysis

Statistical differences between groups were determined using either independent Student's *t *test or one-way ANOVA with a Bonferroni *post hoc *test in the SPSS statistical software, version 16.0 for Windows (Chicago, IL, USA). A *p *value of < 0.05 was considered to indicate statistical significance.

## Results

### Acute effects of CL316,243

**Figure 4:**
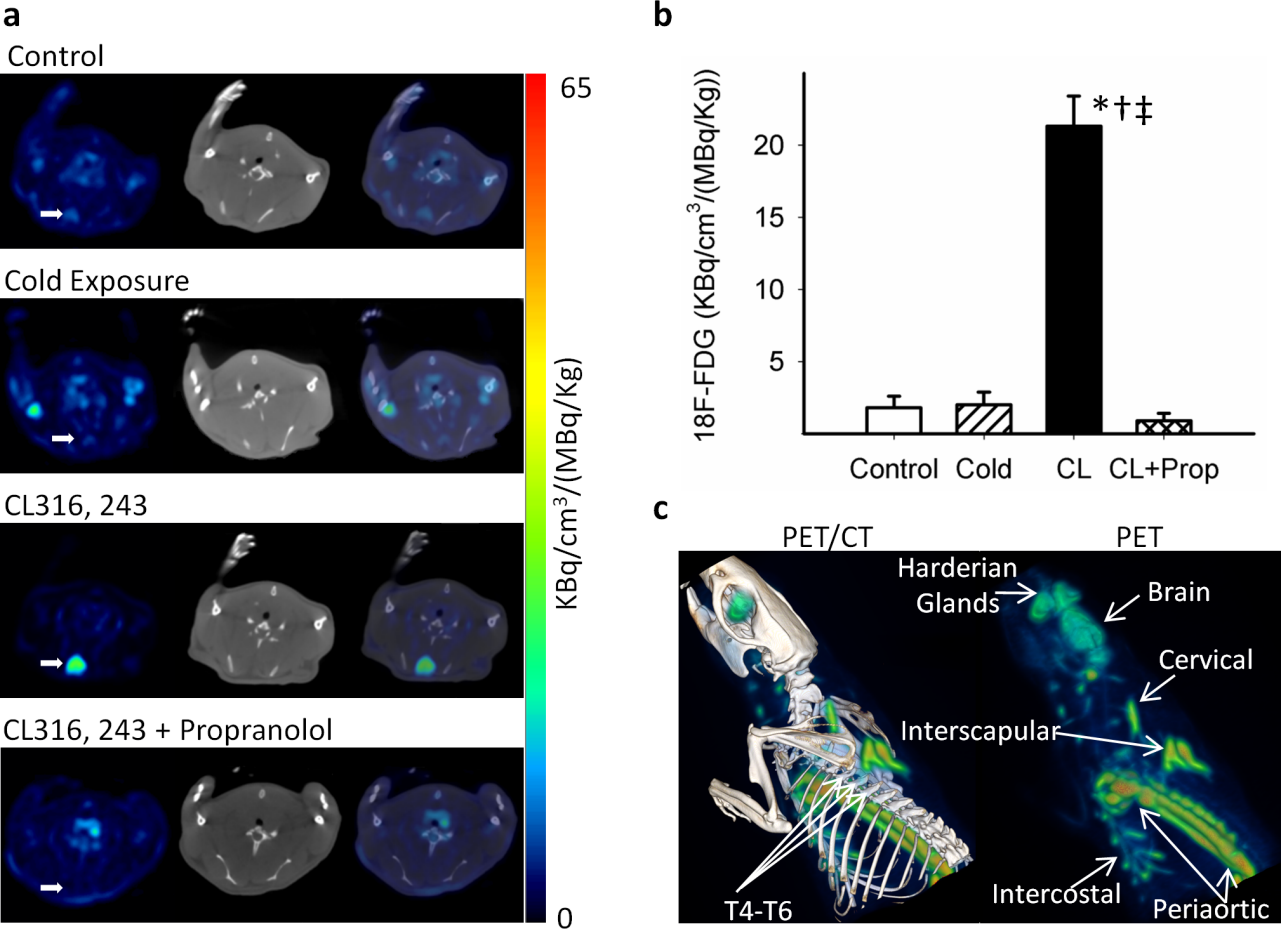
**Transverse views and quantitative and 3-D analyses of PET/CT images**. Transverse views of PET/CT images: PET (left), CT (middle), and fused PET/CT (right) show intense ^18^F-FDG uptake in the CL-activated IBAT, but faint uptakes in the control and the cold-activated IBAT and scarce uptake in the propranolol-treated group (**a**). Quantitative analysis of PET/CT images for IBAT uptake in four studied groups (**b**). Data are mean ± SD; *p *< 0.001 as compared to the control (asterisk), cold-exposed (dagger), and propanolol-treated (double dagger) rats. 3-D analysis of PET (right) and PET/CT (left) images clearly showing interscapular, cervical, periaortic, and intercostal BATs (**c**).

PET/CT images revealed intense ^18^F-FDG uptake in IBAT of CL316,243-treated rats at ambient temperature (Figure 4a). The effect of increased ^18^F-FDG was consistent across animals treated with CL316,243. Treatment of rats with CL316,243 (2 mg/kg) at ambient temperature, 30 min before ^18^F-FDG administration, increased the total ^18^F-FDG uptake of IBAT to 22.7 ± 15.0 kBq/cm^3^/(MBq/kg) which is about 12-fold greater (*p *< 0.001) compared to the ^18^F-FDG uptake in untreated rats of 1.8 ± 0.8 kBq/cm^3^/(MBq/kg). This high amount of ^18^F-FDG uptake in IBAT induced by CL316,243 was decreased dramatically by preinjection of propranolol (reduced by 96.0% to 0.9 ± 0.5 kBq/cm^3^/(MBq/kg), *p *< 0.001). Reduction in the levels of ^18^F-FDG in the propranolol-treated group is consistent with previous findings on the effects of propranolol on BAT (Figure 4b).

### Cold-temperature activation

For purposes of comparison of CL316,243-treated rats at ambient temperature, the rats exposed to cold temperatures also exhibited increases in ^18^F-FDG uptake of IBAT (Figure 4a). However, the increase in the average ^18^F-FDG uptake of IBAT was only 1.1-fold over the control rats at ambient temperature (2.0 ± 0.9 vs. 1.8 ± 0.8 kBq/cm^3^/(MBq/kg), NS; Figure 4b). This increase in ^18^F-FDG uptake is lower when compared to the effects of CL316,243.

### BAT subtypes

Treatment of rats with CL316,243 (2 mg/kg) at ambient temperature increased the total ^18^F-FDG uptake of all regions of BAT significantly. Activated interscapular, cervical, periaortic, and intercostal BATs were clearly observed in a 3-D analysis of ^18^F-FDG PET/CT images, and regions were confirmed anatomically by CT coregistration (Figure 4c; see Additional files 1 and 2). The rank order of ^18^F-FDG uptake in the different BAT areas was found to be IBAT = periaortic > cervical > intercostal (Table 1). This regional distribution was consistent among the different rats treated with CL316,243. In the control rats, the order of ^18^F-FDG uptake was similar but was approximately 10-fold lower. Smaller areas such as the intercostal BAT were difficult to discern under control conditions.

**Table 1:** ^18^F-FDG in different BAT regions in the rat

BAT type	**Control**^**a**^ **(kBq/cm**^**3**^**/(MBq/kg))**	**CL316,243**^**a**^ **(kBq/cm**^**3**^**/(MBq/kg))**	Ratio (CL/control)
Interscapular	1.8 ± 0.8	22.7 ± 15	12.6
Cervical	1.7 ± 0.4	16.3 ± 6.2	9.6
Periaortic	2.5 ± 1.1	23.2 ± 11.6	9.3
Intercostal	NA	9.7 ± 2.2	NA

^a^Mean ± SD, *n *= 3 measured in PET/CT experiments with regions of BAT identified in PET/CT images as shown in Figure 1c; NA, the regions of intercostal BAT in the control animals were unidentifiable due to their small size and very low activity; BAT, brown adipose tissue; CL316,243, (*R, R*)-5-[2-[2,3-(3-chlorphenyl)-2-hydroxyethyl-amino]propyl]-1,3-benzodioxole-2,2-dicarboxylate, disodium salt.

### Volume of IBAT

**Figure 5:**
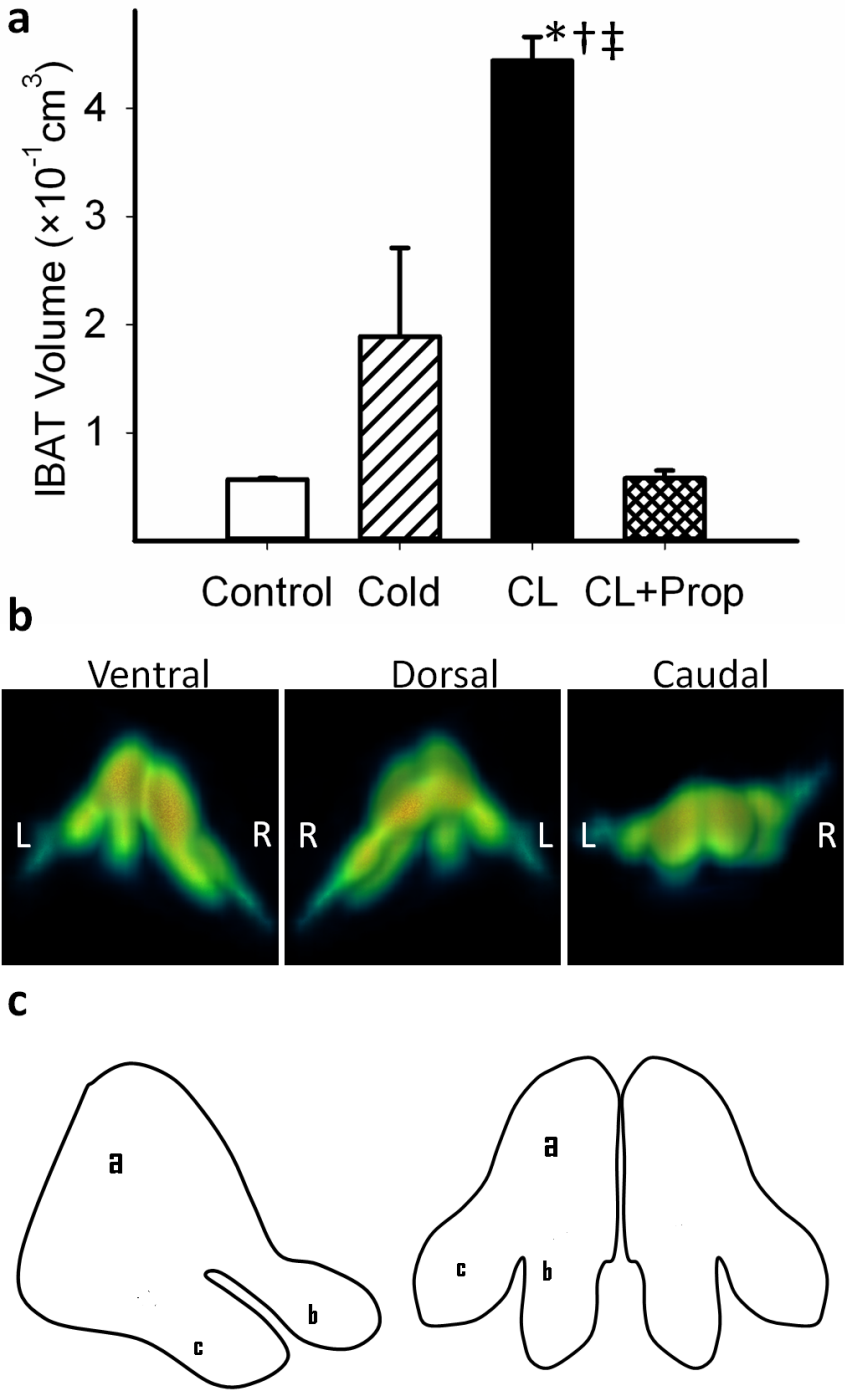
**PET/CT images and their quantitative analysis and the pictorial representation of IBAT**. (**a**) Quantitative analysis of PET/CT images for IBAT volume in the four studied groups. Data are mean ± SD; *p *< 0.001 as compared to the control (asterisk), cold-exposed (dagger), and propanolol-treated (double dagger) rats. (**b**) 3-D PET images of IBAT: ventral, dorsal, and caudal views. (**c**) Pictorial representation of the lateral and dorsal views of IBAT showing a bilateral structure where the medial portions (a) are located near or between T3 and T4 and extends posteriorly and bifurcates (b) and tapers until T6.

The volume of the cold-activated IBAT increased 3.3-fold (0.18 ± 0.08 vs. 0.057 ± 0.001 cm^3^, *p *< 0.001), whereas the volume of the CL-activated IBAT increased 7.7-fold (0.44 ± 0.02 vs. 0.057 ± 0.001 cm^3^, *p *< 0.001) compared to the control (Figure 5a). Propranolol blocked the activation induced by CL316,243 and reduced the IBAT-activated volume. Using 3-D PET image analysis, distinct morphological features of IBAT were discerned in CL316,243-treated rats as seen in Figure 5b. The bilateral multilobular structure of IBAT is shown in Figure 5c with the medial lobe (a) being larger and bifurcating and tapering to two smaller lobes (b and c). This structure of IBAT was seen with approximately a similar profile bilaterally. Based on the ^18^F-FDG uptake of IBAT, greater activation of IBAT begins in the medial parts of the lobe (a) and progresses laterally in the lobes (b) and (c). This morphology of IBAT was consistent in several animals that were treated with CL316,243.

### CL 316,243 dose effects

**Figure 6:**
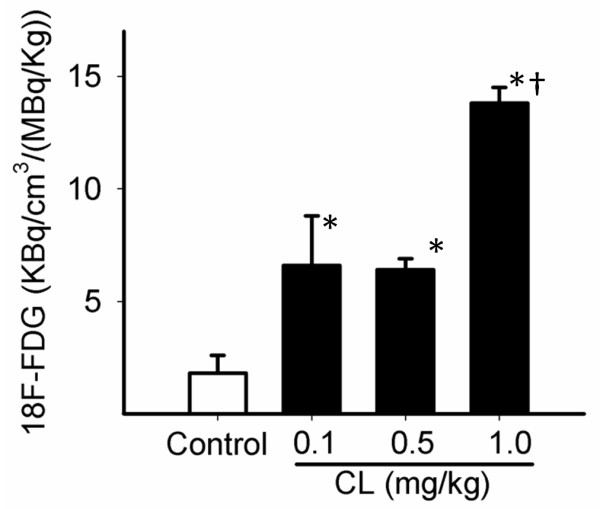
**Quantitative analysis of CL316,243 dose effects on IBAT activity obtained from PET images**. *P *< 0.01 as compared to the control (asterisk) and CL316,243-pretreated (0.1 and 0.5 mg/kg) groups (dagger).

Rats treated acutely with 2 mg/kg of CL316,243 did not show any pharmacological effects and any changes in vital signs. Since chronic studies in rats with lower doses of CL316,243 have been reported, we evaluated a CL316,243 dose-dependent relationship on ^18^F-FDG uptake. Compared to the 12-fold increase by 2 mg/kg, average ^18^F-FDG uptake of IBAT increased 3.6-, 3.4-, and 7.6-fold using doses of 0.1, 0.5, and 1 mg/kg CL, respectively (Figure 6). A fivefold increase in the CL316,243 dose did not significantly affect IBAT ^18^F-FDG uptake.

### Autoradiography

**Figure 7:**
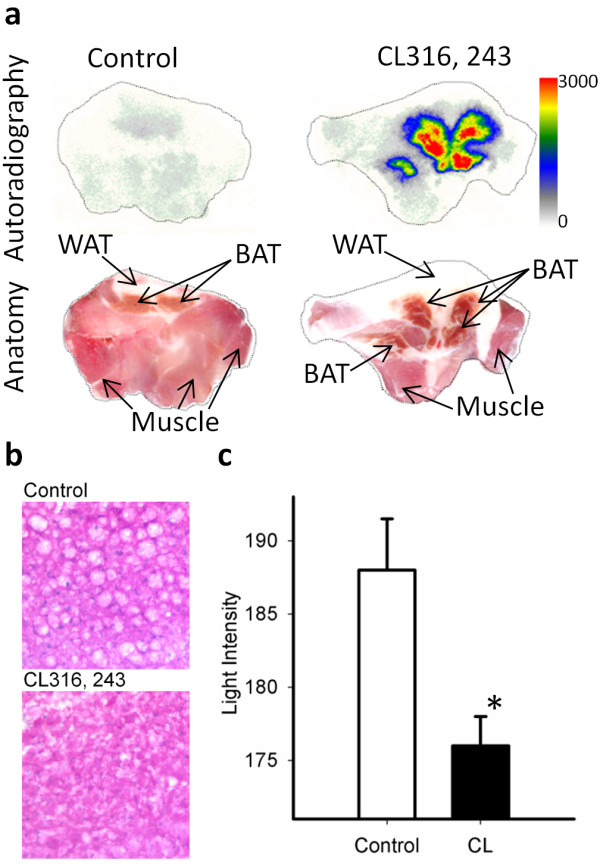
**Autoradiographic images and H&E-stained sections and their quantification**. *Ex vivo *^18^F-FDG autoradiography of BAT (upper row) showing intense uptake in the CL316,243-activated IBAT (right) as compared to the control (left). Consecutive sections of autoradiographic images (lower row) showing IBAT marked by arrows (**a**). H&E-stained sections of control IBAT showing multiocular cells (×200 original magnification) which disappeared when they get activated by CL316,243 (**b**). Quantification of the H&E-stained sections in both groups (**c**). Data are mean ± SD; *p *< 0.004 as compared to the control (asterisk).

IBAT isolated from the control and CL316,243-treated rats had distinct features as seen in Figure 7a. Treatment with CL316,243 induced clear differentiation of large BAT areas compared to the control rats. These BAT areas in CL316,243-treated animals had significant amounts of ^18^F-FDG uptake as seen in autoradiographic images of the transverse sections obtained from the IBAT region. Muscle and WAT areas seen in the histologic consecutive sections showed little ^18^F-FDG uptake. The quantitative data of the autoradiography and autopsy measures in the IBAT and WAT regions are summarized in Table 2. The control animals showed an IBAT/WAT ratio of approximately 3, while the ratio of CL316,243-treated rats was significantly higher, consistent with what is seen in the images in Figure 7a.

**Table 2:** *Ex vivo *measures of ^18^F-FDG in IBAT and WAT

Groups	IBAT		WAT		Ratio (IBAT/WAT)	
	**Autorad**^**a**^ **(10**^**6 **^**DLU/mm**^**2**^**)**	**Autopsy**^**b**^ (kBq/g/(MBq/kg))	**Autorad**^**a**^ **(10**^**6 **^**DLU/mm**^**2**^**)**	**Autopsy**^**b**^ (kBq/g/(MBq/kg))	**Autorad**^**a**^ **(10**^**6 **^**DLU/mm**^**2**^**)**	**Autopsy**^**b**^ (kBq/g/(MBq/kg))
Control	1.6 ± 0.2**	4.3 ± 0.4**	0.5 ± 0.1	1.6 ± 0.1	3.2 ± 0.2	2.7 ± 0.1
CL316,243-treated	13.7 ± 0.1*, **	68.1 ± 18.0*, **	1.1 ± 0.3*	2.4 ± 0.2*	12.5 ± 3.3*	27.6 ± 5.5*

^a^Results of autoradiography in transverse sections of IBAT and the surrounding WAT mean ± SD, *n *= 3; *p < 0.05 as compared to the control; **p < 0.05 as compared to WAT; ^b^Results of counted radioactivity concentrations in autopsied IBAT and the surrounding WAT mean ± SD, *n *= 3; *p < 0.05 as compared to the control; **p < 0.05 as compared to WAT. IBAT, interscapular brown adipose tissue; WAT, white adipose tissue; CL316,243, (*R, R*)-5-[2-[2,3-(3-chlorphenyl)-2-hydroxyethyl-amino]propyl]-1,3-benzodioxole-2,2-dicarboxylate, disodium salt.

### Histology analysis

Staining IBAT of the control animals with H&E showed multiocular cells containing numerous lipid vacuoles which were scarce in the BAT of the CL316,243-treated animals (Figure 7b). The quantitative data of the histology studies showed that the total lipid content of the activated IBAT was significantly reduced (average light intensity 176 ± 2.0 vs. 188 ± 3.5, *p *< 0.004; Figure 7c). There was no significant difference in the WAT lipid content between the two conditions both visually and quantitatively (data not shown).

## Discussion

Our findings support the sympathetic and sensory innervations of BAT where stimulation of β3-adrenoceptors using a selective β3-agonist turns on a cascade of intracellular events ending in hypermetabolism as depicted in Figure 4c and in the Additional files for Figure 4c. In this work, activation of the β3-adrenoceptors using CL316,243 at ambient temperature was able to rapidly stimulate BAT and trigger the uptake of ^18^F-FDG in different regions of the body known to contain BAT. This high degree of ^18^F-FDG enabled the delineation of the extent of different BAT areas including interscapsular, periaortic, cervical, and intercostal BATs. It also provided morphological lobular features of IBAT which consists of bilateral lobes at the midline between the T4 to T6 vertebrae, and each lobe extends and bifurcates laterally (Figure 5b, c). The periaortic BAT was visualized as a large region extending along the vertebral column.

Quantitative increase of ^18^F-FDG in the CL316,243-treated IBAT was more than 12-fold compared to that in the control rats at ambient temperature, and the volume of the activated BAT was over sevenfold compared to the controls. This increase is substantially higher than that of previously reported pharmacological challenge studies using nicotine and ephedrine [13]. Because of the selective nature of CL316,243, it may be inferred that the increase in ^18^F-FDG uptake occurred due to the stimulation of the β3-adrenoreceptor. Propranolol, a nonselective β-blocker, inhibited CL316,243-induced BAT activation to below control levels and confirmed the likelihood of action of CL316,243 via the β3-adrenoceptor.

Activation of BAT by cold exposure has been studied by PET [9]. Long time exposure to cold temperature prior to PET was used to visualize the presence of BAT. However, the difficulty of this method is the variations in sympathetic responses to low temperature in different rats, reducing the reliability of the imaging method. The effect of cold activation on BAT in this study is smaller than that in previous reports [9,11,13] and may be due to the difference in duration and the intensity of cold exposure reported previously (4°C for 4 h).

Our findings of a significant increase in ^18^F-FDG uptake in BAT from a very low dose of CL316,243 (0.1 mg/kg) may suggest a direct role of stimulation of β3-AR. This is consistent with the reported effects of CL316,243 on the overall energy expenditure in BAT [19]. At higher doses, the volume of hypermetabolic BAT increased significantly. Although CL316,243 promotes BAT mitochondrial proliferation, it is unclear if the increases in BAT volume in ^18^F-FDG PET are due to an increase in both brown adipocyte activation and proliferation. Energy expenditure in brown fat is capable of ranging over many orders of magnitude, controlled primarily by sympathetic stimulation. Increases in uncoupled respiration are mediated by rapid changes in UCP intrinsic activity (within seconds), increases in the amount of UCP per cell (within hours), increases in the number of mitochondria per adipocyte (within days), and finally, hyperplasia of brown adipocytes (over days to weeks) [23].

In the early stages of exposure to cold temperatures, mobilization of fatty acids from WAT is also known to be a primary source for the activation of BAT rather than the breakdown of fat depot stored in BAT [24]. Our histology studies showed that the number of lipid vacuoles in BAT was substantially decreased after stimulation by CL316,243, while there was no significant change in the WAT lipid content between the two conditions. Therefore, we believe that in the acute administration of CL316,243, glucose metabolism and lipolysis of stored lipids in BAT are primary sources for the activation of the tissue rather than the mobilization of fatty acids from WAT. Although ^18^F-FDG uptake on PET/CT scans is generally known to represent activation of BAT, one may argue that the observed effect of the agonist might be merely due to a shift in basal BAT metabolism from fatty acids to glucose [25]. Our data confirm an increase in BAT metabolism due to both lipolysis and glucose uptake.

Autoradiography and excised tissue ^18^F-FDG concentration demonstrated that ^18^F-FDG uptake is specific to BAT. Small effects of CL316,243 on WAT ^18^F-FDG uptake were observed. The appearance of brown adipocytes in white fat depots has been recently reported [26]. The origin of these brown adipocytes in WAT is still uncertain, and it is not clear whether these cells develop through differentiation of specific precursors or from white adipocytes. ^18^F-FDG PET/CT is a good imaging tool for measuring small changes in BAT activity and perhaps dispersed brown adipocytes activated by β3-adrenergic agonists or other BAT-activating agents.

## Conclusion

Our results suggest that CL316,243 significantly activates BAT, measurable by ^18^F-FDG uptake at ambient temperature in the rodent model. This is consistent with the β3-adrenoreceptor-mediated metabolic increase in BAT and provides a mechanism to enhance and study BAT activity. β3-AR agonists are potential targets for the pharmacotherapy of obesity and diabetes; however, a better understanding of the mechanisms underlying the metabolism of BAT is needed which will advance management of obesity and diabetes.

## Competing interests

The authors declare that they have no competing interests.

## Authors' contributions

The microPET imaging studies and autoradiographic studies and analyses were carried out by MRM. The microPET/CT data analysis was carried out by CCC. M-LP assisted in the FDG studies. The study and all data acquired were reviewed and the manuscript was coordinated by JM. All authors read and approved the final manuscript.

## Supplementary Material

Additional file 1 **Projection ^18^F-FDG PET image**. Projection ^18^F-FDG PET image of a CL316,243-treated rat showing the BAT, brain, and Harderian glands. Click here for file

Additional file 2 **Projection ^18^F-FDG PET/CT image**. Projection ^18^F-FDG PET/CT image of a CL316,243-treated rat showing the BAT and Harderian glands. Click here for file
